# Transfontanelle photoacoustic imaging for in-vivo cerebral oxygenation measurement

**DOI:** 10.1038/s41598-022-19350-x

**Published:** 2022-09-13

**Authors:** Rayyan Manwar, Laura S. McGuire, Md. Tarikul Islam, Anthony Shoo, Fady T. Charbel, De-Ann M. Pillers, Kamran Avanaki

**Affiliations:** 1 Richard and Loan Hill Department of Bioengineering, University of Illinois at Chicago, Chicago, IL USA; 2 Department of Neurological Surgery, University of Illinois at Chicago – College of Medicine, Chicago, IL USA; 3 Section of Neonatology, Department of Pediatrics, UIHealth Children’s Hospital of the University of Illinois at Chicago, Chicago, IL USA; 4 Department of Dermatology, University of Illinois at Chicago, Chicago, IL USA

**Keywords:** Biomedical engineering, Medical research

## Abstract

The capability of photoacoustic (PA) imaging to measure oxygen saturation through a fontanelle has been demonstrated in large animals *in-vivo*. We called this method, transfontanelle photoacoustic imaging (TFPAI). A surgically induced 2.5 cm diameter cranial window was created in an adult sheep skull to model the human anterior fontanelle. The performance of the TFPAI has been evaluated by comparing the PA-based predicted results against the gold standard of blood gas analyzer measurements.

## Introduction

Brain oxygenation plays a vital role in stabilizing the nervous system so that the entire body can function optimally^1,2^. Particularly in newborns, deficiencies in oxygen content (hypoxia) associated with reduced blood flow (ischemia) to the brain is a major cause of morbidity and mortality^3,4^. Additionally, both preterm and full-term neonates may suffer from secondary injury due to reactive oxygen species formation caused by hyperoxia^5^, an unintended consequence of an intervention, as their endogenous radical scavenging system is immature. Therefore, brain tissue oxygenation or oxygen saturation (sO_2_) represents an important biomarker of neonatal brain health and functionality^4,5^.

Direct monitoring of cerebral blood oxygenation using jugular venous bulb catheters or brain tissue oxygen tension using intracranial oxygen electrodes is invasive and impractical in neonates^6^. The current gold standard for measuring blood oxygenation in neonates is an invasive procedure whereby blood is drawn from arteries, either directly or via an umbilical artery catheter^7,8^. However, these methods cannot measure the cerebral oxygenation directly. Measurements from non-invasive methods, such as oxygen sensors (pulse oximeters) based on optical spectrophotometry, are dependent on superficial capillary beds and cannot be considered as an accurate representation of brain oxygenation^9,10^. There have been promising attempts to quantify brain oxygenation with the use of non-invasive near-infrared spectroscopy (NIRS)^11,12^. This modality has the potential to assist clinicians in assessing changes in cerebral perfusion and oxygenation^13–15^, but it has poor spatial resolution, especially for the small neonatal head. Moreover, NIRS can only measure volume-averaged oxygenation in brain tissue (i.e., unable to distinguish between venous, capillary, and arterial blood), and requires the attachment of cumbersome head caps to accommodate multi-channel fiber optic cables.

Photoacoustic imaging (PAI) is a promising technique that provides noninvasive detection of structural, functional, and molecular anomalies in biological tissue^16^. It combines the technological advances of both optical and acoustic imaging, i.e., the high intrinsic contrast of optical imaging and the spatial resolution of ultrasound imaging^17^. In photoacoustic imaging, nanosecond laser pulses illuminate the tissue at specific wavelengths where endogenous chromophores or exogenous contrast agents have their highest absorption^18,19^. The photon absorption by the absorbing compartments causes a transient temperature change which leads to a localized pressure change that is detectable by an ultrasonic transducer.

Photoacoustic imaging of the brain can simultaneously provide high-resolution images of brain vasculature and tissue oxygenation^20,21^. Previous studies have shown that photoacoustic imaging can measure cerebral blood oxygenation at shallow depths, i.e., at superior sagittal sinus (SSS), of an exposed brain tissue^22,23^, although the validation data was limited. Kang et al.^24^, performed a thorough and systematic analysis of the relationship between photoacoustic-derived sO_2_ in the SSS and that measured directly in the blood samples taken from SSS in piglets. However, in this study venous blood samples were used which does not well represent the brain tissue oxygenation level.

We have developed an imaging system to measure cerebral oxygenation transcranially. Since this device images through a natural skull opening, i.e., fontanelle (or ‘soft spot”, where skull bone formation is incomplete and filled with fibrous membranous tissue), we call it, transfontanelle photoacoustic imaging (TFPAI)^25,26^. We demonstrated the capability of TFPAI to measure brain oxygen saturation on an adult sheep skull model with a surgically induced cranial window in vivo, to represent the average surface area of the anterior fontanelle (2.1 cm) of a newborn. To demonstrate the efficacy of TFPAI in measuring oxygenation, brain regions at various depths and locations were evaluated.

## Materials and methods

**Figure 1:**
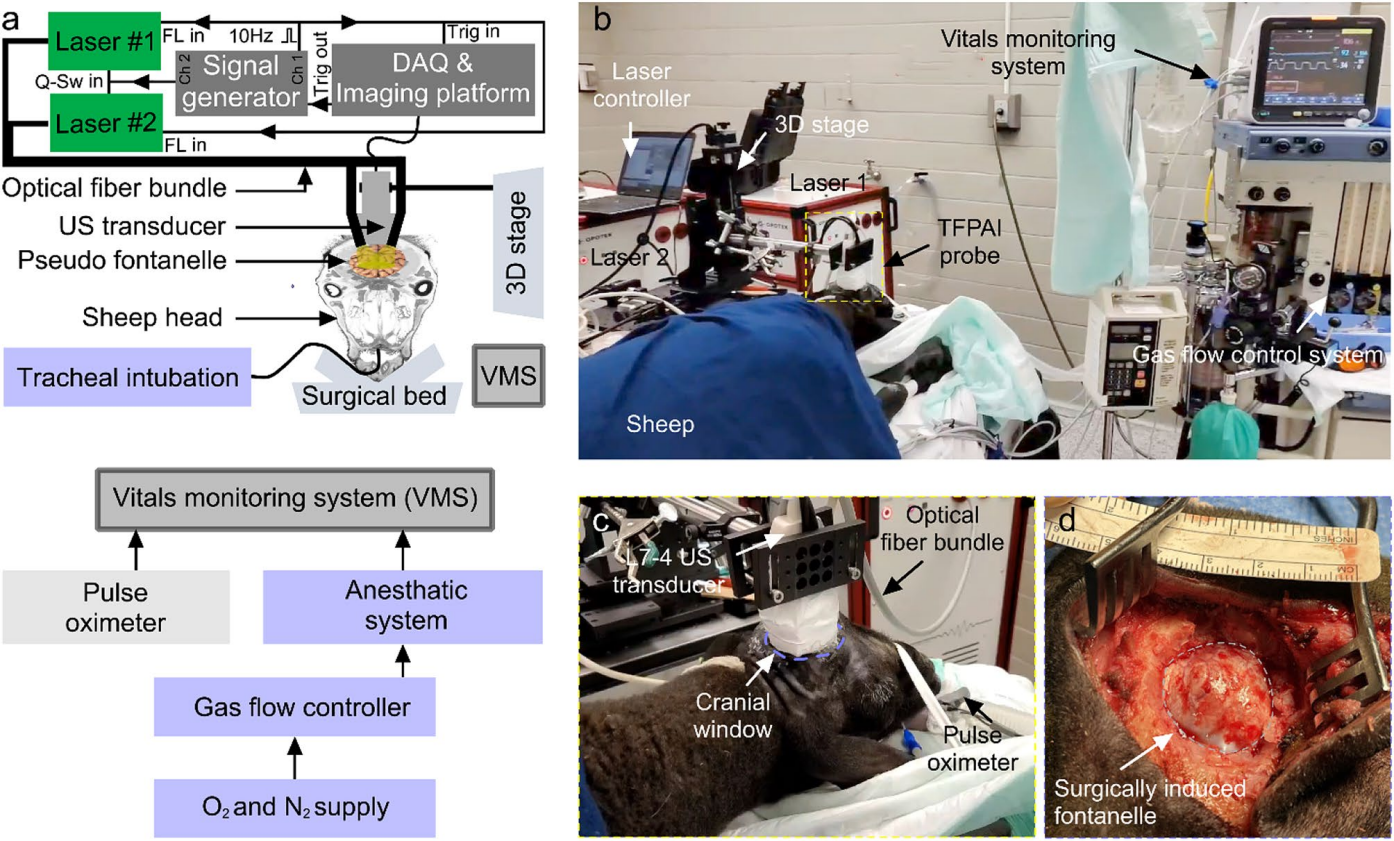
In-vivo brain oxygenation measurement setup using TFPAI. **(a)** Schematic of the experimental setup and process flow of monitoring vitals (prepared in Adobe Illustrator CS6 (version: 16), URL: https://www.adobe.com/products/illustrator.html), **(b)** Key components of the experimental setup for sheep brain oxygen saturation study, **(c)** TFPAI probe configuration and placement over cranial window—a magnified version of the dashed yellow box from (**b**), and **(d)** 2.5 cm diameter surgically induced cranial window representing anterior fontanelle area—gray dashed circular area. Q-Sw: Q-switch, FL: flash lamp, Trig: trigger, DAQ: data acquisition.

A TFPAI probe has been used in vivo on 3 sheep (breed: mix- Katahdin x Dorper, sex: female, weight: 50 kg). We chose sheep because (1) the brain size is closest to a human neonatal brain (especially preterm neonatal brain) and (2) skull anatomy is amenable to surgical access^27–29^. Adult animals do not have a cranial fontanelle and require creation of a “closed” craniectomy in which the cranial opening is covered by the scalp. To mimic the anterior fontanelle, fronto-parietal craniotomy was performed to generate a cranial window. The subject was sedated with acepromazine (0.05 mg/kg) administered 15–30 min prior to placement of an IV catheter (cephalic or saphenous). The animal was placed in a prone position on the operating room table with its head resting on foam and stabilized in position with gel rolls and tape. Ketamine/diazepam (4 mg/kg, 1 mg/kg) was administered via the catheter in order to induce general anesthesia and permit endotracheal intubation. 2–3% Isoflurane was administered using a precision vaporizer and ventilator system (Narkomed GS (Drager, TX, USA)). Anesthetic depth was assessed by monitoring eye position, jaw tone, heart rate, and response to stimulation, with isoflurane dosing adjusted accordingly. The skin was cleansed, and the field prepped and draped in a standard sterile fashion. Using a scalpel, a semi-circular skin incision was made over the temporoparietal regions with the skin flap curvature starting at left frontal bone, extending toward the midline and sagittal suture, and ending above the ear, (see Fig. 1d). Four burr holes in the skull were made using a trepanation bit attached to a surgical electric drill: two burr holes were made near the midline (the sagittal suture), one anterior and another 1 cm posterior; and the other two burr holes were made laterally from the midline 1 cm apart. Using a cylindrical router bit attached to the surgical electric drill, the four burr holes were connected to produce the cranial flap. The resulting rectangular bone fragment was elevated, carefully separated from dura mater, and removed. The dura mater was kept intact to prevent cerebrospinal fluid leak or infection. Overall, an approximately 2.5 cm diameter cranial window was generated. After careful inspection of the craniotomy window for signs of bleeding, additional hemostasis was implemented. A Penrose drain inserted, and the skin flap replaced over the cranial opening and sutured closed; the drain was removed in 1–2 days. Suturing was conducted in layers using absorbable sutures for subcutaneous tissue and 3–0 monofilament (Ethilon) suture for the skin. Proper evacuation of air or fluid from under the skin flap was verified before placing the final skin sutures. This study was approved by the Office of Animal Care and Institutional Biosafety (OACIB) and Institutional Animal Care and Use Committee (IACUC) at the University of Illinois at Chicago.

For the imaging sessions (see Fig. 1a), illumination was provided by two Q-switched Nd:YAG Opotek Phocus HE MOBILE lasers (OPOTEK, LLC) with pulse widths of 5 ns and repetition rate of 10 Hz. The laser was tuned using a dedicated internal optical parametric oscillator (OPO). The target was illuminated by the lasers through two bifurcated fiber bundles (each consists of 414 silica fibers, 200 µm core diameter, and 130 cm length) with a numerical aperture of 0.22, manufactured by Fiberoptics Technology Inc. (Pomfret, CT, USA). On the laser side, there was a 5.5 mm diameter stainless-steel ferrule with an active aperture of 5.0 mm, and on the imaging target side there were two 22 mm × 0.80 mm rectangular bifurcated metal plates with an active aperture of 17 mm × 0.64 mm, secured to the fixture by a 3D-printed holder at 10^◦^ angle from the transducer probe (see Fig. 1b). Fluence, from each laser, at the scalp equaled 30 mJ/cm^2^ at 750 nm which the total energy is still below the ANSI limit^30^. We selected 4 wavelengths, 750 nm, 758 nm, 798 nm, and 850 nm. 750 nm and 758 nm were selected as they accurately measure deoxygenated blood concentration from veins where the absorption coefficient of deoxyhemoglobin (HbR) is an order of magnitude higher (1405 cm^−1^ and 1560 cm^−1^ respectively) than that of oxyhemoglobin (HbO_2_) (518 cm^−1^ and 574 cm^−1^). 850 nm wavelength was selected to accurately measure the oxygenated blood flowing through arteries because the absorption coefficient of HbO_2_ is higher (1058 cm^1^) than that of HbR (691 cm^−1^). 798 nm (isosbestic point) wavelength was used as a reference where the absorption of HbR and HbO_2_ are close to each other (807 cm^−1^ versus 782 cm^−1^). For PA signal detection, a L7-4 linear array (Philips Healthcare, TN, USA) ultrasound probe with 128-elements and 5 MHz central frequency was used (element size: height, 7 mm; width, 0.25 mm), giving a penetration depth of ~ 4 cm into the brain tissue. Both the optical fiber and the probe position were fixed with clamps (see Fig. 1c) and attached to a motorized 3-axis stage for relative positioning and avoiding motion artifacts. PA signal acquisition was performed using a 128-channel, high-frequency, programmable ultrasound system (Vantage 128, Verasonics Inc.). The data acquisition (DAQ) system and the laser flash lamps were triggered using a 10 Hz square pulse train at 5 V peak-peak supplied by a function generator. The Q-switches were triggered by the Vantage 128 trigger output port. All procedures were controlled through a MATLAB 2020b (Mathworks, CA, USA) graphical user interface. B-mode images were generated using Verasonics Inc. proprietary reconstruction algorithm.

3 sheep were participated in this study. During the imaging session, each sheep was anesthetized with 1.5–2.0% isoflurane and kept in a prone position. Oral-endotracheal intubation was used for the delivery of a gas mixture consisting of isoflurane (2–3%) with oxygen (~ 20%) and nitrogen (~ 77%) to the animal. During the imaging sessions, we continuously monitored the animal’s vital signs using Mindray Passport 12 (iPM12 Vet, Mindray Bio-Medical Electronics Co., Ltd., Shenzhen, China). This includes continuous pulse oximeter (sO_2_), electrocardiogram, temperature, respiratory rate, end tidal carbon dioxide measurement. Blood pressure was measured using a catheter inserted into femoral artery, while the pulse oximeter was attached to the tongue. Heart rate and cardiac rhythm were monitored by electrocardiography.

Before positioning the TFPAI probe, the sheep wool above the cranial window was carefully removed to expose the skin layer. A thin layer of ultrasound gel was applied to ensure acoustic coupling between TFPAI probe and the skin layer. Ultrasound B-mode images were utilized as guidance to accurately position the TFPAI probe over the cranial window.

**Figure 2:**
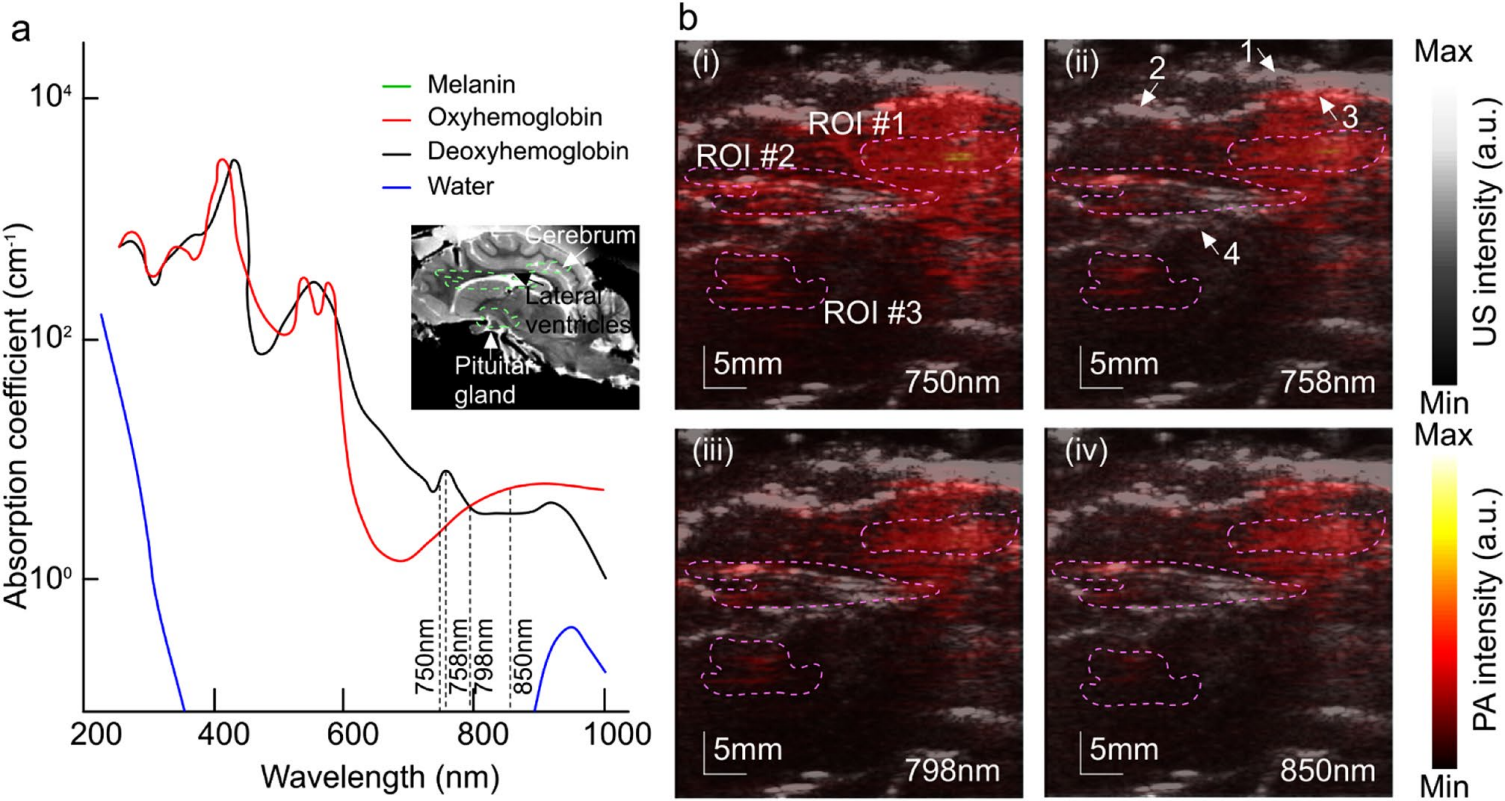
Absorption coefficient spectra of endogenous tissue chromophores and reconstructed PA images overlaid on ultrasound image. (**a**) HbO_2_ and HbR, 150 g/L in blood; water, 80% by volume in tissue; lipid. Figure adapted with from^31^. In the inset, an MRI cross-sectional (sagittal plane) of *ex-vivo* sheep brain depicting major anatomical structure and relative ROI locations^32^ (**b**) ROIs considered for estimating sO_2_ from reconstructed PA images overlaid on US imaging plane at (i) 750 nm, (ii) 758 nm, (iii) 798 nm, (iv) 850 nm. Anatomical structures annotated in (ii) 1: skull, 2: fibrous tissue growth within the cranial window, 3: superficial vasculature, 4: lateral ventricle. (Prepared in Adobe Illustrator CS6 (version: 16), URL: https://www.adobe.com/products/illustrator.html).

We changed the blood oxygenation (sO_2_) in the range of 65% to 100% by varying the fraction (10%, 12%, 14%, 16%, 18%, 20%, 97%) of inspired oxygen (FiO_2_) in the inhaled gas. To generate the gas mixtures varying from 10 to 97% inspired oxygen, we combined medical grade oxygen and nitrogen using precision flow meters to produce a total gas flow rate of 2L per minute to provide an adequate flow for anesthesia. To avoid any movement of the animal affecting the location of imaging, the arterial blood sample was collected from femoral artery and blood gasses were measured immediately before PA signal acquisition to maintain data integrity. Before the main experiment, we performed three complete cycles of varying inspired O_2_ concentrations to determine flow rate, time, and calibration curve between FiO_2_ and sO_2_ from the pulse oximeter. At each oxygenation level, we consecutively acquired photoacoustic images at 750, 758, 798, and 850 nm (Fig. 2a). For three sessions, we acquired a total of 144 B-mode images (2 images for 4 wavelengths at 6 different FiO_2_ and 3 sessions).

Based on the structural/physiological information we obtained from the ultrasound images, three specific regions of interests (ROIs) on each of the PA images were selected to extract the sO_2_. Given the known absorption coefficients of HbO_2_ and HbR, concentrations of the oxygenated and deoxygenated hemoglobin, and in turn sO_2_ were calculated from the PA images using the linear spectral fitting model. Before acquiring images at each FiO_2_, sO_2_ was measured using blood gas analyzer (BGA) (OPTI 4, IDEXX Vetstat, USA) for performance evaluation. At each oxygenation level, the median sO_2_ value was calculated among the 9 sets (3 ROIs × 3 trials) (see Fig. 2b) of the calculated sO_2_ from PA images and compared with the corresponding measured sO_2_ by gold standard BGA. Given the known absorption coefficients of HbO_2_ and HbR, concentrations of oxygenated (C_HbO2_) and deoxygenated hemoglobin (C_Hb_) were computed from the PA images based on the linear spectral fitting model [1–4], and sO_2_ was calculated as:

1 $$sO_{2} (x,y) = \frac{{C_{{HbO_{2} }} (x,y)}}{{C_{{HbO_{2} }} (x,y) + C_{HbR} (x,y)}} \times 100\%$$

## Results and discussion

**Figure 3:**
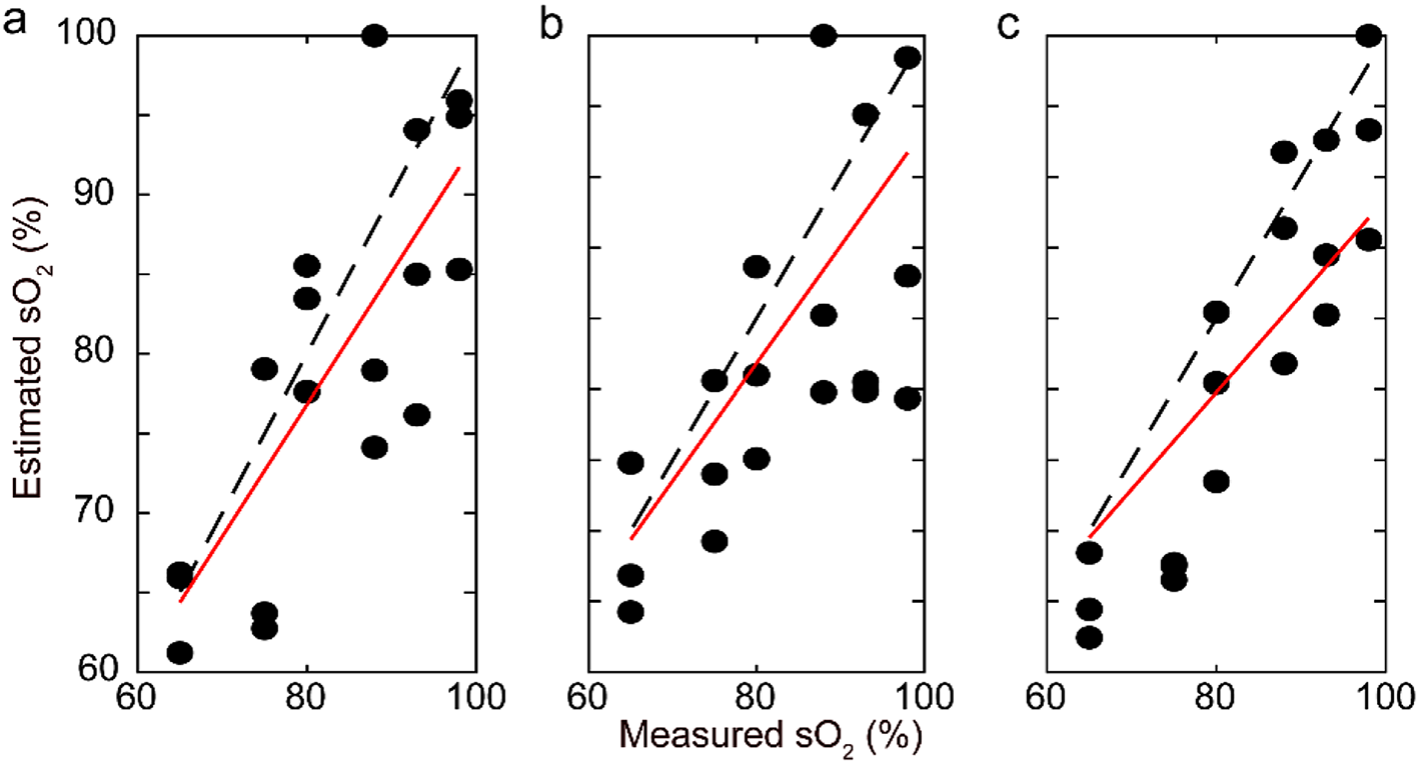
Correlation between estimated (from PA images) and measured (blood gas analyzer) O_2_ saturation for all trials, **(a)** ROI #1, **(b)** ROI #2, and **(c)** ROI #3. Dashed line: arterial blood gas analyzer, Solid line (red): linear fit of estimated sO_2_ from PA images.

A sample set of reconstructed PA images, overlaid on their corresponding US images, acquired at 750 nm, 758 nm, 798 nm, and 850 nm with selected ROIs to estimate the sO_2_ are shown in Fig. 2b. ROI #1 was considered 1 cm deep inside the brain tissue, near the parietal lobe. Oxygenation measurement within ROI #1 is important to understand the extent of the ventricular hemorrhage into the gray matter (periventricular leukomalacia) and white mater injury^33,34^. ROI #2 consists of vasculature surrounding lateral ventricles, 2 cm deep to the fontanelle. This includes the germinal matrix in neonates which can easily be ruptured due to immature formation and initiate Grade 1 and 2 intraventricular hemorrhages^35,36^. ROI #3 was considered near the pituitary gland around 3 cm from the brain surface which will provide insight of pituitary disfunction due to traumatic brain injury in neonates^37,38^. Since, superficial vasculatures (e.g., SSS and cortical veins) carry deoxygenated blood and moreover, PA estimated values are compared to arterial samples (fully oxygenated), we did not consider any ROI near the cortical surface. The linear regression between the estimated and actual sO_2_ is computed for each ROI, the results are shown in Fig. 3a–c. The slope differences from the actual sO_2_ were 0.05, 0.22, and 0.07 for ROI #1, #2, and #3, respectively. The R^2^ values are 0.87, 0.65, and 0.47.

**Figure 4:**
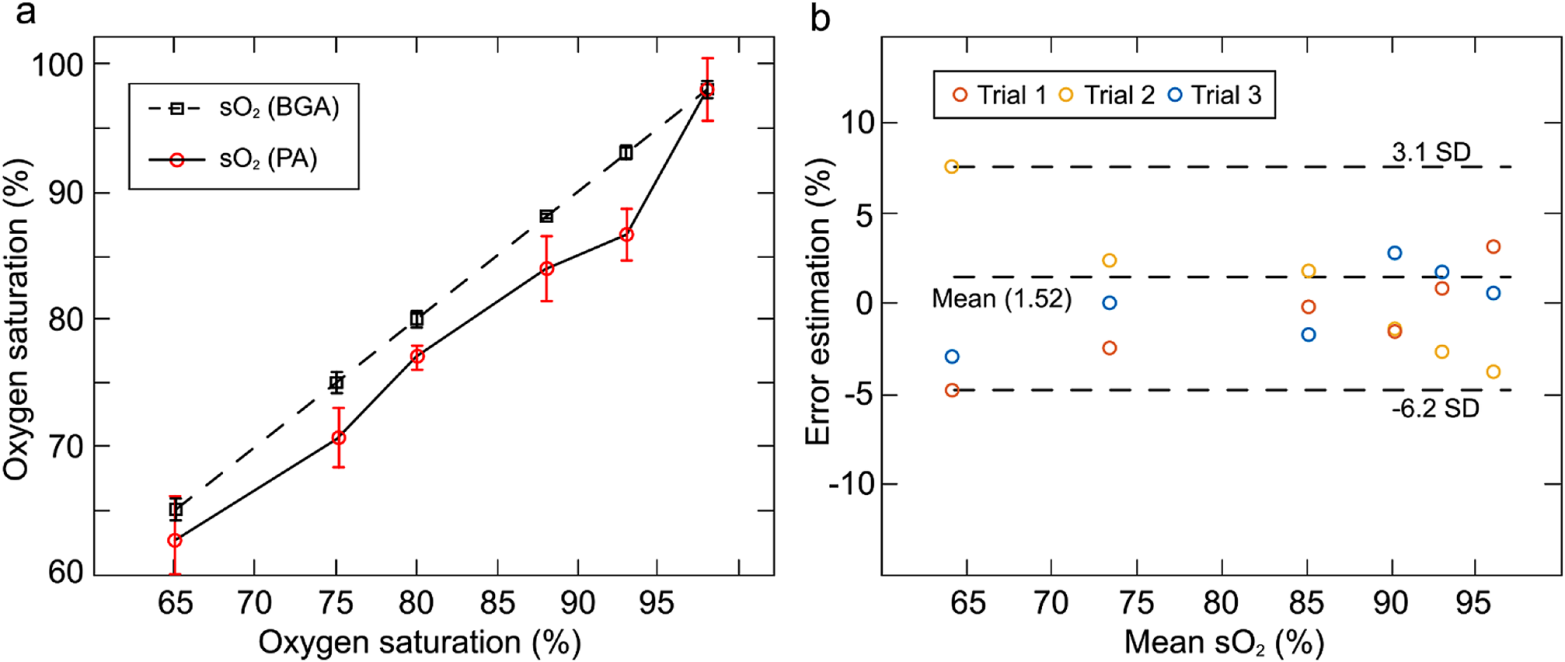
Photoacoustic Oxygen saturation (PA-sO_2_) validation. **(a)** PA estimated sO_2_ plotted against measured sO_2_ from blood gas analyzer. Error bars represent standard deviation (SD) among the trials 1–3, **(b)** Bland–Altman estimation analysis plot showing error estimation for all trials. SD: standard deviation.

The performance of the TFPAI probe in estimating sO_2_ was calculated based on the median of the calculated sO_2_ values from the three trials (Fig. 4a). TFPAI tends to measure lower sO_2_ values with an average deviation of 3.42% from the readings from BGA. For comparison, such deviation between the readings of a pulse oximeter and BGA is 4.12%. The mean estimated error and the 95% confidence interval of the mean error between the two measurements (actual and PA estimated) of sO_2_ for all the trials are shown in Fig. 4b. The PA based estimated sO_2_ exhibited limited error in all three ROIs. Minimum error of 0.34% was observed in cerebral normoxia range (75–90%), followed by 0.64% in cerebral hyperoxia range (90–100%), and 0.83% in hypoxia range (65–75%), as compared to systemic arterial sO_2_.

One of the main experimental challenges was the drift in the pulse oximeter reading during data acquisition, especially when the animal was in the hypoxia range, i.e., with a slight change of FiO_2_ (< 10%) a rapid decay of the oxygenation level occurred. Hence, we had to set the lower limit of FiO_2_ to 10% and minimize the time of data acquisition that was allowed to store two B-mode images at each wavelength.

A lower sO_2_ estimation by TFPAI compared to BGA reading might be related to the slight deviation from the assumed anatomical location^39^. This issue can be seen in the difference between the estimated sO_2_ at ROI #1 and #3, estimated sO_2_ trend (Fig. 3a,c) exhibits an increment of deviation from the gold standard. The main reason might be the existence of venous vasculatures with deoxygenated blood in the vicinity of the assumed anatomical regions; for instance, in Fig. 2b, PA intensity is higher at deoxyhemoglobin-dominant wavelengths (750 nm and 758 nm) as compared to the other two wavelengths. Also, can be seen, in ROI #2 (around the lateral ventricles) exhibited more accurate trend, especially in normoxia range (Fig. 3b). Nevertheless, in all ROIs, the slope was close to 1 and a reasonable linear fit was obtained.

Trial 3 was performed 4 weeks after creating the cranial window when a substantial layer of fibrous tissue growth beneath the scalp may cause the higher deviation from the gold standard as compared to Trials 1 and 2 which were performed 2 weeks from the surgery. This linearity demonstrates the tracking capability of brain oxygenation trends through the pseudo fontanelle. Bland–Altman analysis^40^ indicates no significant bias and a 95% confidence limit of error shows that single point measurements at specific oxygenation levels are reliable (Fig. 4). Overall, the correlation between the PA estimated oxygenation results and the BGA measurements were robust even in the hypoxia range where inspired oxygen was limited to 10%.

## Conclusion

Tissue oxygenation is an important biomarker of neonatal central nervous system health and functionality. However, there are no reliable techniques to directly measure this biomarker. Here, we demonstrated the capability of non-invasive PA imaging using a handheld probe to measure oxygenation through a fontanelle for various conditions, such as hypoxia and hyperoxia *in-vivo*. A pseudo fontanelle was created in an adult sheep, and PA imaging was performed through the scalp.

Spectroscopic analysis was performed based on four carefully chosen wavelengths to extract the oxygen saturation at different locations within the brain tissue. The performance of TFPAI was evaluated and compared to the gold standard blood gas analyzer measurement from arterial blood samples. Superficial structures were not considered in PAI-based sO_2_ estimation to avoid the influence of signal generated by scalp. We observed a strong correlation between the predicted and actual measurement. Inherently, oxygen saturation varies depending on the location where the sample under test is collected. In this study, the oxygen saturation levels were compared to arterial blood samples taken from the femoral artery which explains the deviation between actual and predicted results. TFPAI could measure sO_2_ at deep structures because (i) the light/acoustic attenuation at the cranial window was lower than skull, and (ii) illumination was maximized by simultaneous operation of two lasers, reaching to the total energy of 140 mJ at 750 nm wavelength while maintaining the fluence below the ANSI limit. The results demonstrated here showed the efficacy of the TFPAI probe in determining the oxygenation level. Our next objective is to utilize the probe on neonates with an actual fontanelle. Towards that goal, at first, we plan to further refine the probe capability by developing a calibration technique based on machine learning to compensate for the deviation predicted between cerebral sO_2_, umbilical and capillary sO_2_. We expect that the TFPAI probe will be a valuable monitoring tool especially for neonates with critical conditions and admitted into intensive care unit (NICU).

## In-vivo animal use

The animal used in this study was sourced from Beef and Sheep Research Field Laboratory at University of Illinois at Urbana-Champaign—College of Agricultural, Consumer and Environmental Sciences.

The study protocol was approved by Institutional Animal Care and Use Committee (IACUC) at the University of Illinois at Chicago.

The study was carried out in compliance with the ARRIVE guidelines.

All methods were carried out in accordance with relevant guidelines and regulations.

## Data Availability

The datasets used and/or analyzed during the current study are available from the corresponding author on reasonable request.

## References

[1] Zauner A, Daugherty WP, Bullock MR, Warner DS (2002). Brain oxygenation and energy metabolism: Part I—biological function and pathophysiology. Neurosurgery.

[2] Amann M, Kayser B (2009). Nervous system function during exercise in hypoxia. High Alt. Med. Biol..

[3] Farzam P (2017). Shedding light on the neonatal brain: probing cerebral hemodynamics by diffuse optical spectroscopic methods. Sci. Rep..

[4] Sola A (2014). Safe oxygen saturation targeting and monitoring in preterm infants: Can we avoid hypoxia and hyperoxia?. Acta Paediatr..

[5] Reich B, Hoeber D, Bendix I, Felderhoff-Mueser U (2016). Hyperoxia and the immature brain. Dev. Neurosci..

[6] Zhong, W., Ji, Z. & Sun, C. in *Healthcare.* 1104 (Multidisciplinary Digital Publishing Institute).

[7] Organization, W. H. *WHO guidelines on drawing blood: best practices in phlebotomy*. (World Health Organization, 2010). 23741774

[8] Dukić L, Milevoj Kopčinović L, Dorotić A, Baršić I (2016). Blood gas testing and related measurements: National recommendations on behalf of the croatian society of medical biochemistry and laboratory medicine. Biochemia medica.

[9] Lemmers PM, Toet MC, van Bel F (2008). Impact of patent ductus arteriosus and subsequent therapy with indomethacin on cerebral oxygenation in preterm infants. Pediatrics.

[10] Dix LML, van Bel F, Lemmers PMA (2017). Monitoring cerebral oxygenation in neonates: An update. Front. Pediatr..

[11] Delpy D, Cope M (1997). Quantification in tissue near–infrared spectroscopy. Philos. Trans. R. Soc. London Ser. B: Biol. Sci..

[12] Strangman G, Boas DA, Sutton JP (2002). Non-invasive neuroimaging using near-infrared light. Biol. Psychiat..

[13] Bucher HU, Edwards AD, Lipp AE, Duc G (1993). Comparison between near infrared spectroscopy and 133Xenon clearance for estimation of cerebral blood flow in critically ill preterm infants. Pediatr. Res..

[14] Edwards AD (1988). Cotside measurement of cerebral blood flow in ill newborn infants by near infrared spectroscopy. The Lancet.

[15] Gopinath SP (1995). Early detection of delayed traumatic intracranial hematomas using near-infrared spectroscopy. J. Neurosurg..

[16] Demene C (2017). Functional ultrasound imaging of brain activity in human newborns. Sci. Transl. Med..

[17] Osmanski B-F, Pezet S, Ricobaraza A, Lenkei Z, Tanter M (2014). Functional ultrasound imaging of intrinsic connectivity in the living rat brain with high spatiotemporal resolution. Nat. Commun..

[18] Wang LV (2008). Tutorial on photoacoustic microscopy and computed tomography. IEEE J. Sel. Top. Quantum Electron..

[19] Yang J-M (2012). Simultaneous functional photoacoustic and ultrasonic endoscopy of internal organs in vivo. Nat. Med..

[20] Gamelin J (2009). A real-time photoacoustic tomography system for small animals. Opt. Express.

[21] Yao J (2013). Noninvasive photoacoustic computed tomography of mouse brain metabolism in vivo. Neuroimage.

[22] Petrov I (2012). Optoacoustic monitoring of cerebral venous blood oxygenation though intact scalp in large animals. Opt. Express.

[23] Petrova I (2009). Noninvasive monitoring of cerebral blood oxygenation in ovine superior sagittal sinus with novel multi-wavelength optoacoustic system. Opt. Express.

[24] Kang J (2018). Validation of noninvasive photoacoustic measurements of sagittal sinus oxyhemoglobin saturation in hypoxic neonatal piglets. J. Appl. Physiol..

[25] Avanaki, K. & Gelovani, J. G. Ultrasound and multispectral photoacoustic systems and methods for brain and spinal cord imaging through acoustic windows. Wayne State University. U.S. Patent Application 16/566,212 (2020).

[26] Manwar R, Islam MT, Ranjbaran SM, Avanaki K (2022). Transfontanelle photoacoustic imaging: ultrasound transducer selection analysis. Biomed Opt Express.

[27] Mallard C, Vexler ZS (2015). Modeling ischemia in the immature brain: how translational are animal models?. Stroke.

[28] Stevenson, D. K., Benitz, W. E., Sunshine, P., Hintz, S. R. & Druzin, M. L. *Fetal and neonatal brain injury*. (Cambridge University Press, 2017).

[29] Williams CE, Gunn AJ, Mallard C, Gluckman PD (1992). Outcome after ischemia in the developing sheep brain: An electroencephalographic and histological study. Ann. Neurol..

[30] America, L. I. o. (American National Standards Institute, Inc Washington, DC, 2014).

[31] Yao J, Wang LV (2014). Sensitivity of photoacoustic microscopy. Photoacoustics.

[32] Perentos N (2016). An EEG investigation of sleep homeostasis in healthy and CLN5 batten disease affected sheep. J. Neurosci..

[33] Huang J (2017). Association between perinatal hypoxic-ischemia and periventricular leukomalacia in preterm infants: A systematic review and meta-analysis. PLoS ONE.

[34] Back SA (2017). White matter injury in the preterm infant: Pathology and mechanisms. Acta Neuropathol..

[35] Szpecht D, Szymankiewicz M, Nowak I, Gadzinowski J (2016). Intraventricular hemorrhage in neonates born before 32 weeks of gestation—retrospective analysis of risk factors. Childs Nerv. Syst..

[36] McCrea HJ, Ment LR (2008). The diagnosis, management, and postnatal prevention of intraventricular hemorrhage in the preterm neonate. Clin. Perinatol..

[37] Auble BA (2014). Hypopituitarism in pediatric survivors of inflicted traumatic brain injury. J Neurotraum.

[38] Kurtoğlu S, Özdemir A, Hatipoğlu N (2019). Neonatal hypopituitarism: Approaches to diagnosis and treatment. J. Clin. Res. Pediatr. Endocrinol..

[39] Tajima G (2016). Correlation between regional cerebral oxygen saturation (rSO2) and arterial blood gas (ABG) during cardiopulmonary resuscitation. Circulation.

[40] Giavarina D (2015). Understanding bland altman analysis. Biochemia medica.

